# Role of personality traits for entrepreneurial intentions of young entrepreneurs: A case study of higher education institution

**DOI:** 10.3389/fpsyg.2022.1010412

**Published:** 2022-10-03

**Authors:** Yuanyuan Cao, Muhammad Mujtaba Asad, Lu Wang, Aisha Naz, Norah Almusharraf

**Affiliations:** ^1^School of Economics and Management, Xi’an University of Posts and Telecommunications, Xi’an, China; ^2^Department of Education, Sukkur IBA University, Sukkur, Pakistan; ^3^Applied Linguistics Department, College of Humanities and Sciences (CHS), Prince Sultan University, Riyadh, Saudi Arabia

**Keywords:** entrepreneurial intentions, personality traits, young entrepreneurs, qualitative study, Pakistan

## Abstract

Pakistan being a young country is struggling to provide employment opportunities. However, entrepreneurship is a perceived strategy for reducing unemployment. The trend of entrepreneurship is also emerging among university students. Thus, the ratio of entrepreneurial intention and start-ups is also increasing among university students and graduates. Therefore, this study aims to examine the role of personality traits in the entrepreneurial intentions of young entrepreneurs. Considering this, qualitative methodology was employed with the case study as the research design. A single case of a university with three different departments was taken. In total, *n* = 9 entrepreneurs were purposely selected from business (*n* = 3), computer science (*n* = 3), and education (*n* = 3) departments. The data were collected using semi-structured interviews and documentary analysis of their ventures and success stories. This study followed research ethics, including volunteer participation, confidentiality, and reciprocity. The collected data were analyzed using interpretative phenomenological analysis. The findings reveal three main themes: the desire to be an entrepreneur, learning attitude, and personality traits are the leading factors in the entrepreneurial intentions of students and graduates. However, within these themes, the effect of personality traits (consistency and determination, discipline and locus of control, and risk-taking and tolerance) is found to lead to entrepreneurial intentions among young entrepreneurs. This study concludes that most entrepreneurs believe that the role of personality traits is evident in entrepreneurial intentions. Moreover, the personality traits are further strengthened with entrepreneurial experience and help continue entrepreneurship.

## Introduction

Entrepreneurship is an essential aspect of the business world for economic advancement (Cunningham and Lischeron, 1991; van Praag and Versloot, 2007). Entrepreneurship plays a decisive role in the economic growth of a country (Fritsch and Schindele, 2011; Urbano et al., 2019). Cao and Shi (2021) provided an insight that entrepreneurship uplifts the development of the community, region, and country economically and industrially by generating employment opportunities. Nowadays, the most crucial element of any nation is assumed to be economic stability (Boudreaux et al., 2019; Klofsten et al., 2019). Entrepreneurs produce many jobs for the jobless to reduce the curve of unemployment (Klofsten et al., 2019; Cao and Shi, 2021; Khan et al., 2022). In this way, China has advanced in entrepreneurship by creating links with select and regressive countries to boost its economy and create entrepreneurial opportunities (Ahlstrom and Ding, 2014). Thus, with entrepreneurs’ help, developed nations always orchestrate various awareness campaigns to ensure their netzines and generations secure their future (Malecki, 2018). However, Cohan (2012) stated that the marginalized group of youth could not access education, employment, and other opportunities. Thus, Pakistan has certain constraints that hinder youth from developing their business, especially those who lack self-awareness. Entrepreneurs have transited the economic tracks by providing innovative products and services.

Pakistan is a developing country and is considered a youthful country with 62% of literacy rate of Pakistani youth (Pakistan Bureau of Statistics, 2017), with a high level of unemployment of 6.0% (Trading Economics, 2015; Statista, 2020), which itself is more than neighboring nations, namely, India, Iran, and Bangladesh. The unemployment rate is increasing with an increase in graduates who cannot get desired employment. However, the literacy rate of Pakistani youth is 63.3% (MOFEPT [Ministry Of Federal Education and Professional Training], 2022). Marginalized people have less or no access to education and employment opportunities (Cohan, 2012). Supporting the younger generation in starting their businesses is a strategy to combat unemployment (Ghio et al., 2016; Mubanga et al., 2019).

An entrepreneur is an individual who entrenches or establishes a new business by bringing innovation, bearing most of the risks, and then enjoying rewards (Yan and Guan, 2019). In other words, the entrepreneur is one who seeks to take risks to generate profit and achieve an end goal (Roundy, 2017). The process of setting up a new business is called entrepreneurship. According to Global Entrepreneurial Monitor (GEM [Global Entrepreneurship Report], 2011), 43% of the country’s population can start a business, which is much higher than the other countries (Bangladesh - 24%, Malaysia - 31%, and Turkey - 42%). Conversely, only 23% of Pakistanis want to start a business since most of those, 35%, are afraid of failing (GEM [Global Entrepreneurship Report], 2011). Entrepreneurship is universally believed to play a significant role in a nation’s economic development, improving profitability, creating employment, and providing technological incubation advances (Ghura et al., 2017; Awang et al., 2019). Pakistani youngsters are interested in becoming self-employed and can do so. Self-employment is more prevalent among youth than among adults. About 45% of the young population prefers self-employment compared to adults (OECD and European Commission, 2014). Young generations are also more likely to be their boss and consider working for them a viable option. Compared to adults, 41% of youth believe they will be self-employed soon (OECD and European Commission, 2014). According to the Global Entrepreneurship Monitor, the current statistics of Pakistan show that entrepreneurial activities are increasing; early-stage entrepreneurial activity is 3.7, and established business ownership is 4.72.

Entrepreneurship and enterprise developments are important engines driving economic growth and social development (Galindo and Méndez, 2014), employment creation and innovation (Hathaway and Litan, 2014), and technological development in societies (Schumpeter, 1934). It is critical for generating productivity expansion in developing and developed nations (Kuratko, 2005) and rising economies (Manolova et al., 2010). A link between entrepreneurial ambition and personality characteristics, including self-confidence, risk-taking capacity, accomplishment needs, and perceived control, influences entrepreneurship decisions (Johnson, 1990; Bonnett and Furnham, 1991). On the other hand, a person is encircled by the diversity of social, cultural, socioeconomic, governmental, demographic, and technical influences (Nicotra et al., 2018). As a result, such situational elements can be separated from personal attributes. Some situational factors also influence the preference for entrepreneurial intentions (Stam, 2015; Khan et al., 2022). According to Hisrich (1990), people can be pushed or pulled by situational elements relevant to their personal history and current situations. In a broader sense, entrepreneurship is influenced by cultural and institutional contexts (Wennekers and Thurik, 1999; Khan et al., 2022). Therefore, this study aims to examine the role of personality traits in the entrepreneurial intentions of young entrepreneurs and how do personality traits influence the entrepreneurial intentions of young entrepreneurs in Pakistan.

## Literature review

### Entrepreneurship

Entrepreneurs bring capital change and contribute to the economic growth of a country (Urbano et al., 2019). It is imperative to develop individuals’ interest in new entrepreneurship ventures as a driving force for creating jobs and economic stability. Successful entrepreneurs distinguish themselves from unsuccessful entrepreneurs by interacting with their internal and external loci of control (Gaddam, 2008). Some essential personality traits for entrepreneurial orientation are the need for achievement, locus of control or self-efficacy, and innovativeness; risk-taking is a vital personality attribute, whereas experience, mental ability, and knowledge are important human capital attributes (Frese, 2009).

In recent decades, governments and policymakers are focusing greater attention to entrepreneurship, encouraging and directing a new workforce to tackle economic problems (Merrill et al., 2008; Kim, 2018). Globally, many research studies have focused on determining the factors that can predict entrepreneurial orientation to achieve goals relating to economic growth. Personality traits have been one of the most important streams of research regarding the entrepreneurial intentions of individuals (Kerr et al., 2018). Personality traits are significant factors that have a reliable influence on entrepreneurs. The following section further elaborates on personality traits regarding entrepreneurial intentions.

### Entrepreneurial intentions

A mindset precedes an intention. Passion is a direct precursor and a complete mediator between attitudes and behavioral intentions. Its scope is broad and associated with new ventures planning and execution (Kautonen et al., 2015). Intention is the primary force for generating a behavior (van Gelderen et al., 2018). According to Bruyat (1993), an entrepreneurial intention is akin to a desire. Fayolle (2000) defined it as a will to act to achieve the desired goal. However, according to Bird (1992), an entrepreneurial intention is equivalent to liberty and, therefore, a will to fulfill the visioned goal. Adding to it, such an intention is a frame of consciousness that guides a person’s perspective, attentiveness, experimentation, and actions toward a destination (Bird, 1992). Although the vision is constantly inspiring, it requires focus and determination to bring it to life. According to Bird (1988), it is all about the intention that aids in achieving the organizational objectives. In addition, entrepreneurial intentions are proven predictors of the behaviors and traits of entrepreneurs (Fayolle and Linan, 2014; Delanoe-Gueguen and Linan, 2019). Also, it is emphasized that entrepreneurial intentions are based on the entrepreneur’s requirements, attitudes, behaviors, and beliefs (Hajer and Habib, 2013).

According to Chang et al. (2014), entrepreneurial qualities relevant to entrepreneurial intention can enhance entrepreneurial behavior. However, Frese (2009) and Gorgievski et al. (2018) stated that personality traits could predict attitudes toward entrepreneurship. According to Rauch and Frese (2007) and Kerr et al. (2018), psychological attributes related to entrepreneurship include locus of control, proclivity to take risks, need for achievement, tolerance for uncertainty, and innovativeness. Likewise, Frese and Gielnik (2014) supported that the need for achievement, internal locus of control, tolerance for ambiguity, and risk-taking are usually inclined drivers of entrepreneurial intention. Also, achievement, innovativeness, locus of control, and self-confidence predicted entrepreneurial attitudes (Sánchez, 2013).

### Personality traits

Personality traits can generally be described as the consistent individual reaction caused by external conditions or the environment. Based on the analysis, it is generally believed that the entrepreneurial success of an individual depends on personality traits because traits determine a person’s behavior and decision-making, thereby influencing success. Entrepreneurs with powerful traits have higher performance and run their businesses long term (Ciavarella et al., 2004). On the other hand, entrepreneurs who face initial setbacks lack the desire to continue the engagement.

#### Locus of control

A person’s locus of control is the degree of control over his life. The more significant internal locus of control determines the career path and higher entrepreneurial intentions for successful businesses. It is also the belief of people to control the environment through their actions. People with a higher locus of control will take a risk and establish successful businesses (Gurol and Atsan, 2006). They believe in their actions to control the environment. People with a higher score on a feeling of control usually have clear visions for their future. Entrepreneurs display more excellent intentions when their internal locus of control is more vital (Kristiansen and Indarti, 2004). The internally controlled people successfully show their interests and positively overcome crucial encounters. They efficiently manage social interaction by themselves, rather than relying on people.

#### Need for achievement

Behavioral disposition enables individuals to proceed with certain activities. Following a standard, one can self-evaluate during a challenging event that demands capabilities and desire to accomplish the activities. The need for achievement is also described as the desire and ambition to be successful (Gurol and Atsan, 2006). People with stronger, greater desires and ambitions for success are more likely to become successful entrepreneurs (Orman, 2009). There is a positive correlation between the need for achievement and entrepreneurial behavior. People choose complex and challenging tasks, take responsibility for accomplishments, and expect feedback (Rauch and Frese, 2007).

In need of achievement, the expectations to perform the tasks are better than other entrepreneurs or even own previous performances (Hansemark, 2003). The motivation for achievement increases and develops with the competition with the structure of the desire for change. Therefore, the need for achievement changes and develops over time with newly learned characteristics.

#### Risk tolerance

In the discussion of entrepreneurship, taking calculated risks is a critical approach. Entrepreneurs need to calculate the risk of taking action and evaluate its advantages and disadvantages at all stages. At the same time, they must have the art of tolerating the risks more than other people. Taking a risk and tolerating it is an essential trait of entrepreneurs. The attitude of entrepreneurs in taking risks differentiates them from those of managers and employees. In the entrepreneurial phenomenon, the risk-taking propensity is handling risk, and uncertainty is a common trait. People take risks on alternatives with lower chances, but the alternative result may emerge (Tang et al., 2012). They decide in uncertain situations and utilize them to maximize. Risks may range from business, family, career, to other areas. Risks are essential to entrepreneurial activities because entrepreneurial work is itself a risk.

#### Innovativeness

Innovativeness is one of the essential traits of entrepreneurs. Innovativeness can be described as developing and implementing new ideas with the transition of time in a context. It is a disposition to engage with new ideas to create new things with different characteristics than the existing things and practices (Wiklund and Shepherd, 2005). As a process, innovation results in realization of new ideas and promotes innovative events. For succeed in business, an entrepreneur must be an innovative thinker and doer. The entrepreneur should grasp the opportunity for new products and ways of solving problems. This characteristic brings transition in the business and distinguishes the entrepreneur from ordinary people.

Furthermore, the innovativeness of the entrepreneur develops from the strategic orientation and environmental stimuli (O’Regan and Ghobadian, 2005). Innovation is about creating new value, and this process involves both ideas and knowledge. Innovation is the key to survival of organizations (Gurol and Atsan, 2006).

### Personality traits and entrepreneurial intention

Establishing one’s enterprise necessitates planning and preparation; thus, entrepreneurship is deliberate and planned conduct (Bird, 1992; van Gelderen et al., 2018). Intention has long been a predictive factor in entrepreneurial tasks (Astuti and Martdianty, 2012). According to Kautonen et al. (2015), entrepreneurial intentions are a significant determinant of future entrepreneurial activities. The need for achievements, locus of control, risk-taking, ambiguity tolerance, and innovation are five psychological dimensions that directly affect the entrepreneurial intentions of entrepreneurs (Hornaday and Aboud, 1971; Ahmed et al., 2022). Multidisciplinary and analytical reasoning influences entrepreneurial goals. Business strategies, opportunity analyses, and purpose-oriented behaviors have all been based on cognitive and social aspects of entrepreneurial intentions (Boyd and Vozikis, 1994; Liu et al., 2021). Entrepreneurial ambitions are centered on developing a strategic plan, acquiring resources, and engaging in actions guided by goals. Entrepreneurial goals are also shaped by specific pre-set visions, socioeconomic status, and personality traits of entrepreneurs (Ahmed et al., 2022).

Meanwhile, entrepreneurship begins with a desire to succeed. Entrepreneurial intents are influenced by different loci of control, each of which has its own set of variables (environment, market, finance, and regulations). According to Krueger et al. (2000), entrepreneurs’ contemplation generates aspirations, and such people emphasize the most important process of entrepreneurial intentions. However, according to Autio et al. (1997), emotional and social factors influence young adults’ entrepreneurial inclinations. They also discovered that purposeful factors such as world views, interests, affections, and thoughts generate significance in carrying out desired entrepreneurial intentions. Therefore, personality traits directly influence entrepreneurial intentions along with entrepreneurial behaviors, individuality, surroundings, and demographics (Gaddam, 2008). The literature review found a contextual gap because a significantly smaller number of studies have been conducted in the Pakistani context, specifically in Sindh. Also, the methodological gap has been identified because most studies are conducted quantitatively. However, thorough investigation of young entrepreneurs—their struggles, perceptions, and personality traits—is less.

## Methodology

The study design was qualitative because the aim was to examine the role of personality traits in entrepreneurial intentions because the qualitative research paradigm aids in gaining a thorough grasp of the phenomena under examination from the point of view of individuals conducting the research (Mey, 2022). The real-life environment offers a better opportunity to learn about phenomena in their natural settings, allowing the researcher to gain a profound grasp of genuine experiences (Creswell and Creswell, 2017). This study used a case study design in qualitative research because case studies allow researchers to investigate processes and practices shaped by certain beliefs and practices (Yin, 2009). It also enables the collection of multiple sources of information on the research participants’ experiences (Yin, 2009). Furthermore, according to Creswell and Miller (2000), it is a valuable method to investigate and comprehend any social phenomenon, including individuals, acts and circumstances, and organizations. Thus, this study is a case study with a single case of a well-reputed University of Sindh.

### Research participants

A well-reputed university in the context of Sindh was taken as a single case. This semi-government university comprises five different departments and offers multiple undergraduate and graduate degree programs. Along with curricular activities, it provides students opportunities to organize various co-curricular and extra-curricular activities. Dice Vice and Hult Prize are some examples of entrepreneurial events and activities and platforms for engaging students in entrepreneurial opportunities. The participation of business, education, and computer science departments is evident in entrepreneurial activities. About 10–12 students and graduates have started their businesses. Thus, the sample of *n* = 9 students and graduates, *n* = 3 from each department, is selected through the purposive sampling technique. Purposive sampling was used to choose participants for the study, as shown in Table 1. Purposive sampling is the deliberate selection of samples based on the study requirements. According to Creswell (2003), purposive sampling helps comprehend the research issue. The researcher selects participants based on their willingness to participate, competency, and knowledge about the subject of interest. The individuals’ experiences were also considered. The selection criteria were (a) the participant belongs to business, education, and computer science departments, (b) the participant has started his/her business, and (c) the participant has at least 1 year of experience in starting and running his/her own business. Considering this, *n* = 9 entrepreneurs were purposely selected for data collection.

**TABLE 1:** Participant selection criteria.

Case	A Well-reputed University of Sindh, Pakistan		
Departments	Business	Computer science	Education
Participants	*N* = 3	*N* = 3	*N* = 3
Gender	All Males	All Males	All Males
Nature of Ventures	Food, Restaurants, and Saloons	State Agency, Software Houses, and E-Commerce	Physical and Virtual Academies, Testing Services, Notes For Test-Preparations

The case study approach allows researchers to acquire data using various tools. So, the researchers employed an interview guide and document analysis for semi-structured interviews and focus group interviews, document analysis, and field observation. Each technique aided researchers in gathering data from various sources and allowed collecting a large amount of data from many perspectives, which is the main activity of qualitative research (Creswell and Poth, 2016). The data were collected using in-depth semi-structured interviews and document analysis of the entrepreneurs’ success stories. The interview guide was developed by the researchers based on the reviewed literature. The interviews were analyzed using interpretative phenomenological analysis because the study aimed to examine the role of personality traits of young entrepreneurs in leading them toward entrepreneurial intentions. As the data of lived experiences of initiating and running a business venture were gathered, interpretative phenomenological analysis was suitable to analyze such data. Transcription, familiarization, categorization, emergence, and connections across themes were involved. A researcher ensured all the research ethics are followed during the entire research project. Research ethics refers to rules of conduct, typically conformity to a code or a set of principles.

## Findings

The interpretative phenomenological analysis of entrepreneurs yielded three main themes with sub-themes: (a) desire to be an entrepreneur—be my boss, become an asset, generate employment, and contribute to the economic growth; (b) learning attitude—flexible, reflections, discourse, and innovation; and (c) personality traits—consistency, determination, discipline, locus of control, risk-taking, and tolerance. The desire to be an entrepreneur, learning attitude, and personality traits are the leading factors toward the entrepreneurial intentions of students and graduates. However, within these themes, the effect of personality traits is the cornerstone for leading toward entrepreneurial intentions among youngsters.

### Desire to be an entrepreneur

Under this, three sub-themes were categorized through in-depth interviews and documentary analysis based on commonality with the significant theme. Many factors were found to influence students and graduates toward entrepreneurship. Thus, one of the common aspects among all the participants was shown to have a burning desire to become an entrepreneur. Almost every participant said, “*I am not a kind of 9:00 to 5:00 bound worker following the same routine.”* In addition, one of the participants said, *“I used to see jobholders following the same routine without exponential growth, which triggered me to go for something different but with exponential growth.”* Likewise, one other stated that *“I cannot work with boundaries of time and for short-term.”*

#### To be my boss

The first sub-theme is to become one’s boss. As mentioned earlier, most participants did not favor working for others following their specific set of rules. For this, almost all the participants said, *”I cannot work for other organizations even for more money because it will lead them to get the long-run benefit. However, with my own business, I might earn less but have the satisfaction that the real benefit is of mine either for short or long term.”* However, one of the participants elaborated that *”working with ease and flexibility helps me do my best. I cannot follow others’ instructions, and thus, I do not fit for corporate structure.”*

#### To become an asset

The second sub-theme is to become an asset. It was viewed as the desire not only to work for self but to make innovation and become an asset itself. All the participants claimed, *“With the job, my identity would not have come forward.”* However, one participant said, *”I found hunger and restlessness to do something which makes me and becomes my asset.”* Likewise, others mentioned, *”I want to be known by my venture; it is my ultimate identity.”*

#### Generate employment and contribute to the economic growth

The third sub-theme is generating employment and contributing to the country’s economic growth. Most participants claimed, *”I wanted to help the poor by generating opportunities for them, and with my venture, I provided.”* Similarly, others mentioned, *”Serving my community is the ultimate goal of my life; I want to work for my family and country by generating employment opportunities.”* Considering this, every one of the views that *”Pakistan is suffering financial crisis due to less economic growth and fewer business ventures; however, the role of young entrepreneurs needs to be linked with economic growth of the country broadly.”*

### Learning attitude

Under the umbrella of this theme, three sub-themes can be categorized from in-depth interviews and documentary analysis based on commonality with the significant theme. It was found to be the common aspect among all the participants. Almost every participant said, “*Practical learning of business can only be acquired through initiating and running a business venture.”* In addition, one participant with an academic qualification said, *“I learned all the idealistic things during my business degree program; however, it was different when I went to market. Thus, I would suggest teaching realistic content”.* Likewise, others with no prior qualification related to the business stated, *“It was not my field of a degree program; however, for the success of my venture, I went for several seminars, workshops, and self-study.”*

#### Flexibility toward situational factors

The first sub-theme is to have a flexible attitude toward situational factors. As mentioned earlier, most participants had a burning desire to become entrepreneurs and showed a flexible attitude toward the situational factors before, during, and after their ventures. As one participant said, *”My partner and I belong to a stable family and well-reputed university, yet one of the owners used to remain in the kitchen, and I used to take orders from our customers to manage human resources.”* However, it was also mentioned that *“I used to buy all the stuff by myself, give it an attractive packaging and deliver to my customers in initial days.”* Also, someone highlighted that *”though I belong to a humble family, I managed to teach my students free of cost for about one and a half years, to get it established.”*

#### Reflections and discourses for improvements

The second sub-themes are reflections on the self and business ventures and having a discourse with experts to learn from their experiences. About all the participants claimed, *“the entire entrepreneurial journey was full of learning because I learned from my experiences and mistakes.”* However, one participant said, *”I had run about five different ventures and have experienced many failures, but with failures, I have learned a lot, even from making partnerships to hiring the right persons.”* Also, the others stated that *”we used to sit with other entrepreneurs and discuss internal affairs of business ventures, and we learn a lot from each other.”* Likewise, it was found that almost all entrepreneurs have a monthly meeting with staff to discuss the current practices and decide on future improvements.

#### Innovation to add value

The third sub-theme is innovation to add value to the product and attract the targeted audience. Almost all the participants agreed that *”innovation is the distinctive characteristic of an entrepreneur.”* As one participant said, *”when I was preparing for the entry test of a well-reputed university, I found no relevant material for my preparation. Thus, when I intended to start my business, I targeted that and got a huge response”.* Also, it was mentioned that *”with a thorough search, we found that the ideas of making innovation in café trend, utilizing bank side, and targeting high tea preference of students at universities.”* Similarly, one of the participants said, *”I even moved to different cities and then finalized the idea, innovated it, and launched it with the target of reaching to entire Pakistan.”*

### Personality traits

Under this significant theme, the three sub-themes were categorized through in-depth interviews and documentary analysis based on commonality with the significant theme. All the participants had the common aspect of consistency and determination, discipline, locus of control, and risk-taking and tolerance. Almost every participant said that “*Personality traits play a crucial role in deciding whether a person can become an entrepreneur or not and can run a business or not.”* In addition, one of the participants who had an academic qualification in the business said, *”entrepreneurship is not a term to be given to a person but a state to go through and reach a destination with certain attributes.”* Likewise, the others with another educational background stated, *”it is not the qualification but personality which decides profession, especially of entrepreneurship.”*

#### Consistency and determination

The first sub-theme has consistency and determination in personality, which leads to an entrepreneur’s success. As mentioned earlier, most participants found themselves consistent and determined with their burning desire to become entrepreneurs. For that, they faced several hardships and challenging times, even failures. For this, one participant said, *”I reduced my socialization and gathering time so that I manage time for growing my venture.”* Similarly, one other participant said, *”I am an extrovert, but I feel now I have become introverted due to less socialization, and I can compromise everything for growing my venture.”* Likewise, another participant stated, *”I do not remember I ever finished any task before joining entrepreneurship. It has made me consistent and determinant. Now I always finish all my tasks within time by putting all my efforts”.* Also, it is highlighted by almost all that *”entrepreneurship requires consistency and determination to nurture your venture like that of nurturing a baby. An entrepreneur is the mother of a newborn who has to have nurtured with care and love”.*

#### Discipline and locus of control

The second sub-theme is discipline and locus of control in the personality of an entrepreneur. All the participants claimed, *“There are many challenges from every domain of life in entrepreneurship but having control over them is the only possible solution.”* As mentioned by one of the participants, *”I got hospitalized for more than 3 months, and I was unable to say a single word. However, I managed to control all the stuff and shared all the responsibility with the team and got everything controlled.”* Also, it was mentioned by one of the participants that *”it was my wedding day. I only reached there by evening and left my wife all alone after a day to continue with my stuff, and this way I controlled the entire situation before getting diverted to my new journey of life.”* Similarly, one of the participants said, *”I do not believe on 9:00 to 5:00 schedule. However, I believe in ensuring discipline for running a venture smoothly without losing track”.*

#### Risk-taking and tolerance

The third sub-theme is taking risks and having patience and tolerance to deal with business challenges. Almost all the participants agreed that *”it is difficult to have patience all the time, but it is necessary to have it.”* As one participant said, *“being an entrepreneur, we need to remain patient and have tolerance power along with accepting it that some days are good and some are bad. Not all days bring goods to sell and profit, and sometimes it takes more than months and years to get the profit”.* Also, it was mentioned that *”taking risks without thinking of consequences is as important as tolerating other factors.”* Similarly, it was highlighted by one of the participants, *”I have left two competitive jobs and took the risk and invested in the venture.”* Likewise, others mentioned that *”having an influential family status and working as an entrepreneur was difficult. I got criticism from the majority; I remained patient, tolerated the situation, took risks, and chose to remain an entrepreneur”.*

The successful entrepreneur possesses essential characteristics that require becoming fearless, confident, and robust with self-created ideas, not necessarily being financially stable. They start from less and endeavor hard to get it to where it becomes a distinctive brand and fulfills customers’ needs. So being an entrepreneur is easy but being a successful and exceptional entrepreneur is not smooth sailing but a tough job. It invariably takes much hard work, perseverance, and motivation to get to the point where one can achieve the success one wants. Moreover, successful entrepreneurs attempt to discover opportunities from simple and unexpected things by paying attention to them. There are specific characteristics found essential among entrepreneurs. Vision is the art of seeing what is invisible to others. Vision is the root that enables individuals to ruminate for upcoming calculated risks and equip themselves before building up a new business. Entrepreneurs with solid vision almost succeed once they step out with self-created ideas and implement them to some extent. Likewise, persistence is the key to success; before their enterprise, many entrepreneurs might have difficulty communicating with and incentivizing someone about their products and services. Each of them is greatly inspired and motivated to build up an empire. Indeed, our society is strong enough to make someone feel demoted and demeaned and to prevent an individual from starting a new business; those people are always willing to highlight minor and inconsequential flaws but never endeavor to succor beginners in their new venture. Deep motivation and self-analysis are essential to avoid criticism at that stage.

**FIGURE 1:**
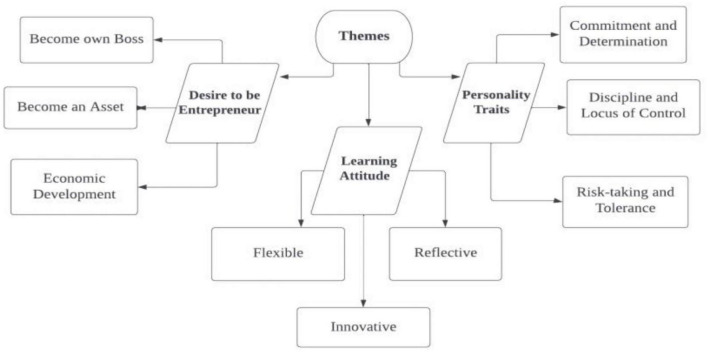
Cumulative themes of entrepreneur characteristics.

Similarly, creativity helps entrepreneurs spot business opportunities in everyday life, as well as passion and patience; if entrepreneurs have the both, they are near their destination. Most people avoid passion and try to leap for shortcuts to become a successful businessman overnight, but unfortunately, they fail to continue to live the same life they used to live. Having said this, an entrepreneur must have specific characteristics to become a renowned entrepreneur. The cumulative themes are shown in Figure 1.

## Discussion

The present study was conducted to explore the role of personality traits in the entrepreneurial intentions of young entrepreneurs. The results of a single case with nine semi-structured interviews and their documentary analysis revealed three major themes related to the personal attributes of young entrepreneurs, which lead to their entrepreneurial intentions. Working under a boss was the most consistent response from all the participants. This could be due to the intelligent working habits of the young generation, which allows them work from anywhere and, at any time, regardless of following local set rules of organization (Bloom et al., 2011; Hawke et al., 2019). Adding to it, it enables an entrepreneur to effectively deal with personal, social, and professional life (Kašperová et al., 2018; Tomaselli, 2019).

Similarly, contributing to the country’s socioeconomic growth is also found to be the leading force for young entrepreneurs and affects their entrepreneurial intention (Bögenhold, 2019). The findings are also supported by Ranjan (2019) and Sergi et al. (2019) that entrepreneurship enhances the economic growth of a country and enables generation of employment opportunities. On the other hand, the sustainability of entrepreneurial intentions is found to be influenced by the learnability of entrepreneurs. The participants showed flexible, reflective, and innovative attitudes toward entrepreneurship, which are valuable aspects of the continuity of entrepreneurship.

According to van Hugten et al. (2021), flexibility is an important personality trait of entrepreneurs that helps them motivate, sustain, and be satisfied with the entrepreneurial decision. Also, the entrepreneurial experience develops a lens of the learner, who learns from diverse experiences and experts through reflections and innovation (Kaseorg et al., 2010). Moreover, personality traits are found to be playing a pivotal role in directing entrepreneurial intentions. Entrepreneurs showed resilience, consistency, determination, discipline, locus of control, risk-taking, and tolerance as the key personality traits that made them entrepreneurs and helped them continue with entrepreneurship in future (Vodã and Florea, 2019). As one participant said, “it is not the motivation but discipline which matters a lot in business”; thus, having a disciplined personality with the balance of all the required traits makes a person entrepreneur. These findings support Karabulut (2016) that entrepreneurial intention is influenced by personality attributes, including locus of control, determination, consistency, risk-taking, tolerance, innovation, and entrepreneurial alertness. Therefore, personal attributes and personality traits influence entrepreneurial intentions and ensure entrepreneurship continuity.

## Research implications

This study has attempted to fill the contextual and methodological gaps in the existing literature. Thus, it has implications for entrepreneurial education to become practical and recommends at least involving students in a single entrepreneurial venture start-up project. Also, the research and practical implication of this study is to further strengthen for developing the attitude of young people toward entrepreneurship based on the entrepreneurial experiences specified in this study. Furthermore, this study will contribute to entrepreneurial intention, which can be influenced by personality attributes, including determination, consistency, and risk-taking, to support their educational needs and expenses as well. The findings of this study can also be used as a reference to design a framework for the youth to become educational entrepreneurs and policymakers to develop a contextual framework for entrepreneurs based on different personality traits of youth and other related contexts in the region.

## Conclusion

As the study aimed to examine the role of personality traits in the entrepreneurial intentions of young entrepreneurs, the analysis reveals that certain personality traits affect the entrepreneurial intentions of young entrepreneurs. The aspects of rigidity toward job setting, learnability, flexibility, and personality traits, including consistency and determination, discipline, locus of control, and risk-taking and tolerance, are significant contributors to a preference for choosing and sustaining entrepreneurial intention. It also highlights the lack of practical entrepreneurial education in Pakistan; however, a few entrepreneurs from other academic backgrounds are also achieving success. This aspect differentiates educational experience from having a desire and certain personality traits to choose and remain in the field of entrepreneurship. Therefore, this study concludes that most entrepreneurs believe that the role of personality traits is evident in entrepreneurial intentions. Also, the personality traits are further strengthened by entrepreneurial experience and help in the continuity of entrepreneurship.

In addition, there are few limitations to this study: the study has only focused on entrepreneurs who have already established their ventures. Meanwhile, the shift of personality traits could be measured from pre- and post-test with an experiment/intervention of a business venture. This could lead to exploring how students become entrepreneurs, regardless of having business-related academic qualifications.

## Data availability statement

The raw data supporting the conclusions of this article will be made available by the authors, without undue reservation.

## Ethics statement

The studies involving human participants were reviewed and approved by School of Education, Sukkur IBA University. The patients/participants provided their written informed consent to participate in this study.

## Author contributions

YC and MA proposed the research idea and analyzed the results. LW, AN, and NA carried out the methodology and extensively edited the manuscript. All authors contributed to the article and approved the submitted version.
